# Parent experiences of the esophageal atresia journey during the early post-natal period: results from a support group perspective

**DOI:** 10.1007/s00431-024-05640-1

**Published:** 2024-06-12

**Authors:** Corné de Vos, Werner de Vos, Anke Widemann-Grolig, Lizelle van Wyk, Daniel Sidler, Pierre Goussard

**Affiliations:** 1 Division of Pediatric Surgery, Tygerberg Hospital, Stellenbosch University and Tygerberg Hospital, Francie Van Zijl Drive, PO Box 19063, Tygerberg, 7505 South Africa; 2 Division of Surgery, Stellenbosch University, Francie Van Zijl Drive, PO Box 241, Cape Town, 8000 South Africa; 3 Esophageal Atresia Global Support Groups (EAT), Sommerrainstrasse 61, 70374 Stuttgart, Germany; 4 Department of Pediatrics and Child Health, Tygerberg Hospital, Stellenbosch University and Tygerberg Hospital, Francie Van Zijl Drive, PO Box 241, Tygerberg, 8000 South Africa; 5 Center for Medical Ethics and Law, Faculty of Health and Sciences, Stellenbosch University, Tygerberg Campus, Tygerberg, 8000 South Africa

**Keywords:** Esophageal atresia, Support groups, Emotional support

## Abstract

Surgically repaired esophageal atresia (EA) is associated with chronic esophageal and respiratory morbidity that require ongoing management. The care of chronically ill children can exert considerable stress on parents, thereby potentially affecting their mental well-being. In response to this, disease-specific support groups have emerged with the aim to bring together individuals facing similar challenges, facilitating the exchange of experiences within a nurturing environment, and providing essential emotional support. In the context of this international collaborative study, we investigated the role played by EA-specific support groups in promoting the emotional well-being of EA families. An anonymous online survey was distributed through a network comprising 12 international EA support groups affiliated with the Federation of Esophageal Atresia and Tracheoesophageal Fistula (EAT) during May and August of 2022. In the study, 96 parents from 23 different countries completed the survey. Fifty-six percent indicated a lack of interaction with other EA families during the initial diagnosis, with 91% expressing the belief that such contact would have been beneficial. Participants exhibited a unanimous recognition of the critical role played by disease-specific support groups in navigating the emotional journey associated with EA.

*Conclusion*: The findings of this global survey align with existing literature, reaffirming the beneficial impact of disease-specific support groups tailored for EA patients and their families on their emotional well-being. These groups provide a valuable platform for the exchange of personal experiences and narratives, delivering advantages to both those who share their stories and those who receive this valuable information.

**Supplementary Information:**

The online version contains supplementary material available at 10.1007/s00431-024-05640-1.

**Table:** 

**What is Known**:
• *Parents are exposed to several stressors during the neonatal period.* • *Emotional well-being of EA families is important.* **What is New:** • *EA support groups play a valuable role in the emotional well-being of EA families.* • *Family-centered care of neonates should also include older siblings of neonates in NICU.*

## Introduction

Esophageal atresia (EA) is a congenital condition that necessitates surgical intervention during the neonatal period often leading to increased morbidity and prolonged hospitalization [1]. EA is associated with chronic esophageal and respiratory morbidity that require ongoing management [2]. The care of chronically ill children can exert considerable stress on parents, thereby potentially adversely affecting their mental well-being [3]. This, in turn, may have implications for the well-being of the entire family [3].

Support groups tailored to specific diseases aim to provide social benefits by alleviating isolation, expanding social networks, and fostering empathetic relationships within the group [4]. Such groups bring together individuals facing similar challenges to share their experiences in a nurturing setting, offering emotional support to both those sharing and those listening. Additionally, support groups share valuable disease-specific resources with its members further assisting them in navigating their specific journeys [5].

In 1984, a parent support group, exclusively focused on EA, was founded in Germany, and has since grown to be one of the largest worldwide [6]. By 2001, 10% of all children born with EA and/or their families in Germany were members of this group, which includes a scientific board of pediatric surgeons convening regularly to address parent and patient-related concerns [6]. More recent numbers have around 20% of EA families in Germany belonging to this group. Inspired by this success, similar EA support groups have emerged worldwide. In 2019, an EA support group was founded in South Africa, following the German model [7].

Insufficient evidence exists regarding the significance of support groups, particularly within the framework for families of patients born with surgical correctable congenital diseases like EA. A study published in 2015 investigated the impact of support groups on patients born with anorectal malformations and demonstrated that these forums provide valuable services to families [8]. The perceived value of these groups includes the exchange of day-to-day information as well as educational opportunities facilitated through social media, networking, and face-to-face meetings [8]. Additional investigations have indicated that parent-led support groups contribute skill acquisition, heightened empowerment, and a sense of communal belonging among parents of children with rare diseases [9].

This international collaborative research project, initiated in partnership between Stellenbosch University (SU) and EAT, aimed to determine the role played by EA-specific support groups in promoting the emotional well-being of EA families, more specifically the parents/caregivers of EA children in the early post-natal period.

## Materials and methods

We employed a cross-sectional descriptive analysis using an anonymous online survey (Appendix 1). This survey was created and distributed via Stellenbosch University Surveys (SUN surveys), a web-based e-survey service [10]. The survey was developed from the literature in conjunction with EAT, after first being piloted in South Africa [7]. The survey was distributed by the consortium comprising all 12 international support groups dedicated to EA, each of which is an affiliated member of EAT. This distribution took place during the months of May and August of 2022.

Members of each of the global EA support groups were invited to complete the anonymous survey. Participation was voluntary and included both patients (older than 18 years) born with EA and their families. A QR code and online link was shared via email, social media, or WhatsApp to members of EAT who in turn send it to their members. The total amount of invites sent was not documented as part of the online survey.

The survey itself was structured into five distinct sections including demographic data, particulars surrounding the child’s birth, initial diagnosis and presence of associated anomalies, information about the time of initial EA surgery, and questions pertaining to parental emotions and support throughout their journey. All participants were required to provide online consent before the start of the survey. The survey contained no open-ended questions and were available in English.

### Statistical analysis and ethical considerations

Data collection was managed by SUN surveys and descriptive statistical analysis was performed and expressed as numbers and percentages. Prior to commencing the study, ethical permission (reference, N21/10/119, S20/10/260) was secured.

## Results

During the months of May and August of 2022, 115 participants followed the online link to start the survey. Of these, 108 participants (94%) from 23 different countries successfully completed the online survey, 96 (89%) of which were parents/caregivers of EA patients. The three countries most participants lived in were the United Kingdom (UK) (34%), followed by South Africa (SA) (27%) and Germany (11%). A comprehensive breakdown of the demographic distribution is visually presented in Table 1. The survey included 12 distinct support groups with TOFS (Trachea-Oesophageal Fistula Support, UK) (30%), OATSA (Oesophageal Atresia and Trachea-oesophageal Fistula association of South Africa) (25%), and KEKS (Kinder und Erwachsene mit kranker Speiseröhre, Germany/Austria) (14%), being the 3 groups most represented.

**Table 1:** Demographic distribution of survey participants according to continent with further distribution specifically for Africa

Continent	Number of participants (*n* = 108)
North America	4
South America	6
Africa*	30
Europe**	63
Australasia	5

^*^Participants from the African continent subdivide into participants from South Africa (*n* = 29) and Kenya (*n* = 1)

^**^Participants from Europe subdivided into participants from the United Kingdom (*n* = 37) and participants from the rest of Europe (*n* = 26)

**Fig. 1:**
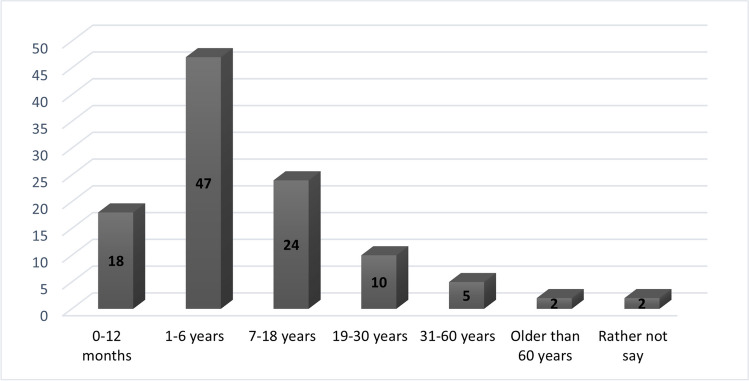
Age distribution of patients born with EA at time of survey completion

The study found that a majority (59%) of the patients born with EA were male, and 45% were born at full term. At the time of survey completion, 44% of the EA patients fell within the age range of 1 to 6 years, as visually depicted in Fig. 1. Among the EA patient cohort, 70% had one or more siblings, and 6% were part of twins. One participant reported a family history of EA in a biological grandparent. Demographics are presented in Table 2.

**Table 2:** Highlights of demographic data collected from the survey

Demographics	*n* = 108 %
Gender of patients born with EA (m:f)	59:40
Gestational age of babies born with EA at time of birth	
• Premature*	39
• Term	53
• Unsure	8
Age of parents at the time of patient born with EA’s birth (%father:%mother)	
• < 19 years old	1:1
• 20–40 years old	85:89
• > 40 years old	10:6
Current relationship status of the parents (%father:%mother)	
• Single parent	1:6
• Married	73:73
• Divorced	2:3
• In a relationship	12:10
• Rather not say	12:7

*EA*, esophageal atresia; *M*, male; *F*, female

^*^Premature, birth prior to 37 weeks gestational age

**Fig. 2:**
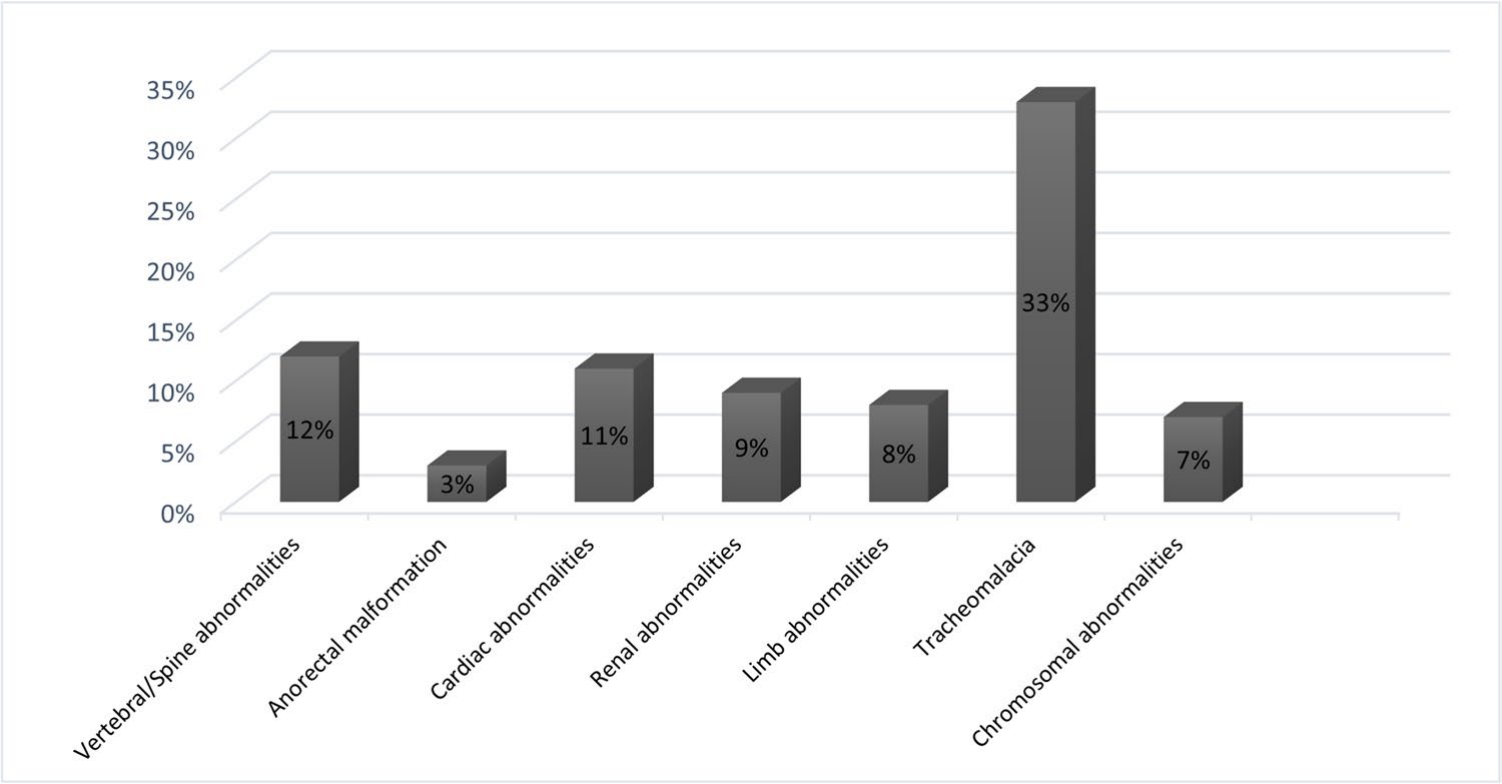
Presence of associated abnormalities

A total 48% of EA patients received a diagnosis of tracheomalacia and 63% were diagnosed with an associated abnormality that included VACTERL anomalies (vertebral, anorectal, cardiac, tracheoesophageal, renal, and limb) and chromosomal abnormalities (Fig. 2).

### Perinatal history

Of the 108 participants, 96 (89%) respondents were parents or caregivers. Among these, 41 (43%) parents or caregivers reported an abnormal antenatal ultrasound finding, of which 22 (54%) of this group reported an antenatal suspicion of EA. Only 7/41 (32%) parents or caregivers received counselling regarding the suspected diagnosis and/or possible outcome following the baby’s birth and surgery. It was unanimously agreed by all parents or caregivers that this counselling proved beneficial, assisting them in better preparing for the forthcoming challenges.

Fifty-five (57%) babies required transfer to a different hospital for their first surgery, and among these cases only 24 (44%) allowed the mother to accompany the baby during the transfer. Most of the babies were placed in a neonatal intensive care unit (NICU), with a mere 15 (16%) of parents or caregivers being granted permission to sleep beside their babies. In 20 (21%) cases, siblings were allowed to visit the baby born with EA, while for the majority (*n* = 60, 63%) only older family members were allowed to visit. A significant number of parents or caregivers (*n* = 66, 69%) expressed a sense of missed bonding opportunities with their newborns due to their initial inability to hold or interact with them.

### Emotional well-being

Exploration into the emotional responses of parents or caregivers upon receiving their child’s initial diagnosis and subsequent surgery revealed that the vast majority (*n* = 89, 93%) experienced feelings of shock, guilt, worry, or a combination thereof. Furthermore, a notable 29 (30%) of the parents of caregivers expressed that they did not fully comprehend the diagnosis.

A substantial majority (*n* = 87, 91%) of parents/caregivers within the survey indicated that they primarily received emotional support from family and friends during this traumatic and emotional time. Fifty-six (58%) reported receiving varying degrees of support from hospital staff, which included nursing staff (79%), medical doctors (55%), social workers (32%), and trauma counselor’s (21%).

Among the surveyed participants, 62 (57%) had been diagnosed with one or more of the following conditions sometime after the birth of their EA child: post-traumatic stress disorder (PTSD) (16%), anxiety (18%), and/or depression (24%).

### The importance of sharing experiences

Fifty-four (56%) of the parents or caregivers who participated in the study reported that they had no interaction with parents or families of other children with EA during the initial diagnosis and hospital stay. Among these, an overwhelming 91% believed that having such contact would have been beneficial during this difficult and emotionally charged time. Moreover, a significant 84 (88%) parents or caregivers felt that sharing the story about their experiences would have assisted them in coping, with an even higher number (*n* = 91, 95%) agreeing that such sharing would also be beneficial to others facing similar circumstances. Nearly all (*n* = 95, 99%) participants concurred on the indispensability of disease-specific support groups, recognizing their value in navigating the emotional journey associated with this rare congenital disease.

## Discussion

This study presents the results of an international collaborative survey in which we participated. The survey focused on assessing the emotional well-being specifically of parents/caregivers of children born with EA, starting in the early post-natal period. An overwhelming majority of the parents/caregivers experienced an emotional time during their child’s initial diagnosis, with less than half reporting that they had contact with other affected families during this time. Nearly all participants believed that sharing stories within a support group setting would benefit others who were new to the experience, and three-quarters felt that sharing the experience would also benefit the sharer. Almost all participants agreed that EA-specific support groups are beneficial in the emotional journey associated with this disease.

Support groups serve as collective platforms with a focused mission at addressing specific challenges, extending mutual assistance, and facilitating the coping process with emotional hardships for individuals who share common challenges [11]. Within these groups, parents not only find disease-related resources, but also a network to receive and offer support, share achievements and setbacks, embrace their children’s conditions, and establish meaningful connections with those navigating similar emotional journeys [5]. Chronic illnesses, including congenital conditions like EA, have the potential to profoundly affect both patients and their families, leading to the emergence to emotional challenges that underscore the importance of timely intervention [12].

Previous research has emphasized the crucial role of psychological support for parents, beginning during the neonatal period and ideally provided by a multidisciplinary team [13]. Parents of neonates admitted to the NICU are exposed to various stressors [14]. Ganguly et al. identified the rapid delivery of information by staff as a significant stressor for parents in the NICU [15]. Similarly, factors such as the fear of communicating with healthcare professionals and concerns regarding access to information about their babies have been identified as added parental stressors, all of which have the potential to significantly affect parental mental health [14]. Other stressors that merit consideration are uncertainty surrounding the neonate’s diagnosis and surgical procedures, cultural practices, financial constraints, and a perceived lack of control [16]. Peer-to-peer support for parents of neonates in the NICU is indeed recognized as a valuable supplement to the services provided by the NICU staff [17]. This form of support offers a platform for sharing experiences without fear of judgment and contributes to reducing the stress and anxiety experienced by NICU parents, fostering a sense of empowerment [17]. The NICU Family Support (NFS) program in the United States exemplifies this approach by enhancing parents’ experience through educational initiatives, hosting of supportive activities, and the establishment of connections between families and NICU staff [18]. An extension of this program is the Family-Integrated Care (FiCare) module that has been introduced to further empower parents as partners in the care of their infant in the NICU [19]. This concept has three levels that includes supporting the parents, interventions delivered by the parents themselves, and parent-partnered neonatal care empowering parents to be part of the hospital care of their ill infant [19]. All these are important aspects relating to the emotional journey during the early neonatal period of parents of children born with rare diseases including EA. The results from our study suggest that there is a need to improve the overall post-natal care for EA families. The FiCare module has been proven to reduce stress and anxiety for parents with neonates in the NICU and can potentially aid EA parents to navigate being discharged from the NICU and assist them with the journey ahead [19].

Our result from this survey highlights several concerning issues related to how medical professionals treat the parents or caregivers of neonates born with EA. Our data suggests that their seems to be a global lack of family-centered care in the management of EA families during the neonatal period. Fifty-seven percent of neonates in our survey were transferred to specialist units, with only 44% of their mothers allowed to accompany them during the transfer. A mere 16% of parents could sleep next to their babies during the NICU admission. Additionally, the vast majority (69%) of the parents or caregivers in our study expressed a sense of missed bonding opportunities with their newborns due to the inability to hold or interact with them during those first few days after birth. The physical closeness is just as important as emotional closeness and crucial to the holistic well-being of both the babies and the parents [20]. Our survey indicates that there is room for improvement in how healthcare professionals involve parents in the care of their newborns. We furthermore recommend prioritizing early bonding experiences for these parents in order to improve their emotional well-being.

In 2013, a study was conducted investigating the role of support groups in children born with anorectal anomalies [8]. They found that the majority of these groups were established by families, and around 80% of them had affiliations with healthcare professionals [8]. This factor alone highlights the possibility to incorporate these groups as part of the holistic approach of complex congenital diseases such as EA.

Another important aspect highlighted by our survey is the limited access that siblings have to their new brother or sister, with only 21% of cases reporting that siblings visited the NICU. Family-centered practices should extend beyond parents to include siblings. In 1983, a study conducted by Schwab et al. found that sibling visits to the NICU have the potential to be beneficial to both the siblings and the families [21]. The arrival of a new baby has been shown, despite its joy, to be a source of stress for older siblings, and this stress can be exacerbated by the infant’s admission to the NICU [22]. Allowing siblings to visit can contribute to a less challenging experience for both the parents and older siblings and has the potential to create a more supportive and nurturing environment for the entire family [22].

The establishment of the first EA support group in Germany in 1984 marked a pivotal moment, leading to the emergence of various other groups worldwide [6]. Schier et al.’s work in 2001 underscores the impact of support groups on mitigating the clinical and emotional long-term consequences of EA [6]. The authors highlighted that such support groups offer patients valuable disease-specific information, alleviating the isolation commonly associated with rare conditions like EA [6]. Notably, the German group played a foundational role in the inception of EAT, an overarching organization uniting multiple EA support groups globally. EAT’s mission resolves around fostering exchanges of experiences and knowledge among patients and healthcare professional and elevating global awareness of EA [23].

The establishment of disease-specific support groups for EA has been pivotal, furnishing crucial information and support, and particularly support that bridges the isolation so often linked to this rare condition. Our study’s participants concurred, recognizing the pivotal role such support groups play in bolstering their emotional well-being. Overall, our findings accentuate the potential benefits of EA-specific support groups in enhancing the emotional health of both patients and their families confronted with this uncommon condition. Healthcare professionals should aim to connect parents of children born with EA with other parents or support groups early during the peri-natal period. This can be achieved by incorporating any of the mentioned strategies in their multidisciplinary approach to treatment of EA families.

Nevertheless, it is essential to acknowledge the study’s constraint of focusing solely on existing group members when interpreting the findings. We do acknowledge that distribution of an English-lonely survey might lead to potential bias and needs to be addressed in future research. An additional noteworthy constraint is the exclusive reliance on an online survey, thereby restricting accessibility and participation solely to individuals with the requisite technological facilities, potentially constraining the participant pool. We also accept that by not identifying specific triggers or events leading to PTSD in parents diagnosed with this disease, as another limitation to this study that should be explored in future research.

## Conclusion

The results of this global survey are in harmony with the existing literature, highlighting the beneficial role that disease-specific support groups, customized for EA families, play in the promoting of emotional well-being. These groups provide a valuable platform for exchanging experiences and narratives, yielding benefits for both those who share their stories and those who receive this information.

However, our presented data raises concerns about the level of family-centered care provided to parents or caregivers of neonates born with EA. Healthcare professionals should consider the emotional and psychological needs of parents and work to improve the support and involvement they offer during the care journey of this group of children.

Given the distinctive challenges associated with rare conditions like EA, it becomes crucial to formally integrate disease-specific support groups into comprehensive multidisciplinary treatment strategies. This integration holds the potential to enhance the long-term outcomes for patients and their families. The robust support network already in place for individuals affected by EA stands as a potential model for other congenital diseases amenable to surgical correction.

### Supplementary Information

Below is the link to the electronic supplementary material.

Supplementary file1 (PDF 353 KB)

## Abbreviations

EA: Esophageal atresia
EAT: The Federation of Esophageal atresia and Tracheoesophageal Fistula
KEKS: Kinder und Erwachsene mit kranker Speiseröhre
NFS: NICU Family Support
NICU: Neonatal intensive care unit
OATSA: Oesophageal Atresia and Trachea-oesophageal Fistula association of South Africa
PTSD: Post-traumatic stress disorder
SA: South Africa
SU: Stellenbosch University
SUN surveys: Stellenbosch University Surveys
TOFS: Trachea-Oesophageal Fistula Support
UK: United Kingdom
VACTERL: Vertebrae, anorectal, tracheoesophageal, renal, limb
