# Supplementary material for: Parent experiences of the esophageal atresia journey during the early post-natal period: results from a support group perspective
Source: Eur J Pediatr. 2024 Jun 12;183(9):3777–83. doi: 10.1007/s00431-024-05640-1 (PMC11322197; doi:10.1007/s00431-024-05640-1)
Supplement: Supplementary file 1 — Supplementary file1 (PDF 353 KB) [file 431_2024_5640_MOESM1_ESM.pdf]

UNIVERSITEIT IYUNIVESITHI STELLENBOSCH UNIVERSITY 100 1918-2018

saam vorentoe · masiye phambili · forward together

EAT support group survey

*

Stellenbosch UNIVERSITY IYUNIVESITHI UNIVERSITEIT

EAT Esophageal ATresia Global Support Groups

SU/EAT Survey: Emotional well-being and support groups.

Before you start.

Participation in this survey is completely voluntary. It is anonymous, and no personal identifiers will form part of the survey.

Only members of a parent/patient support group (official support group or as part as a social media support group of OA/EA e.g Facebook or WhatsApp support groups) may participate in this survey.

All participants need to be 18 years and older.

Data collected will be used as part of a PhD study link to Stellenbosch University, South Africa. The study has been approved by the Health Research Ethics Committee of Stellenbosch University (N21/10/119 and S20/10/260). The results will be made available to EAT and may be used for publications in medical journals and at medical conferences.

Any questions may be directed to any of the study co-ordinators (listed below). Should you need any help with emotional support after this survey, please contact your local medical team or support group for referrals.

Thank you for taking 5 minutes to help us with this.

Principal investigator: Dr C de Vos (cdevos@sun.ac.za)

Co-investigators: Dr W de Vos (werner_devos@yahoo.com), Dr Annemie Burke (5annemie5@gmail.com)

EAT: Anke Weidemann-Grolig (awg@keks.org)

Supervisor: Prof D Sidler

☐ I agree to all the conditions listed in the consent page

*** What is your relationship to the person born with oesophageal atresia (OA/EA) and/or a tracheo-oesophageal fistula (TOF/TEF)? (Born without a complete food-pipe)**

- ☐ Self
- ☐ Parent (Mother or Father)
- ☐ Caregiver or Legal Guardian
- ☐ Grandparent
- ☐ Sibling (Brother or Sister)
- ☐ Other

*** In which country do you currently live?**

____

*** Do you belong to a support group that is part of EAT?**

- ☐ Yes
- ☐ No

If **YES**, to which support group do you belong?

____

If NO (not part of a group that is part of EAT), what is the name of your support group.

____

*** Current age of patient.**

How old is the patient/person born with OA/TOF (EA/TEF) NOW?

- ☐ Infant (0-12 months)
- ☐ 1 - 6 years
- ☐ 7 - 18 years
- ☐ 19 - 30 years
- ☐ 31 - 60 years
- ☐ Older than 60 years
- ☐ None Of The Above

*** What is the sex of the person born with OA/TOF (EA/TEF)?**

- ☐ Male
- ☐ Female
- ☐ Divers
- ☐ Rather not say

*** Details of the MOTHER of the person born with OA/TOF (EA/TEF):**

What was her age at the time of the baby's birth?

- ☐ Younger than 19 years
- ☐ 20 - 40 years
- ☐ Older than 40 years
- ☐ Rather not say
- ☐ Unknown

*** Details of the MOTHER of the person born with OA/TOF (EA/TEF):**

Current relationship status:

- ☐ Single parent
- ☐ Married
- ☐ Divorced
- ☐ In a relationship
- ☐ Rather not say
- ☐ Do not know
- ☐ Other

*** Details of the FATHER of the person born with OA/TOF (EA/TEF):**

Age at the time of baby's birth?

- ☐ Younger than 19 years
- ☐ 20 - 40 years
- ☐ Older than 40 years
- ☐ Rather not say
- ☐ Unknown

*** Details of the FATHER of the person born with OA/TOF (EA/TEF):**

Current relationship status:

- ☐ Single parent
- ☐ Married
- ☐ Divorced
- ☐ In a relationship
- ☐ Rather not say
- ☐ Do not know
- ☐ Other

*** Siblings.**

How many siblings does the child/person born with OA/TOF (EA/TEF) currently have?

- ☐ 1
- ☐ 2
- ☐ 3
- ☐ 4
- ☐ More than 4
- ☐ No siblings

*** Twins.**

Is the person born with OA/TOF (EA/TEF) one of a twin?

- [ ] Yes
- [ ] No

Antenatal (During the pregnancy)

Did the doctors expect anything when you/your wife/your mother was pregnant?

- [ ] Yes
- [ ] No
- [ ] Unsure

Antenatal (During the pregnancy)

If YES, did they expect an OA/TOF (EA/TEF)?

- [ ] Yes
- [ ] No
- [ ] Unsure

*** Family History**

Is there anyone else in the family that was born with an OA/TOF (EA/TEF)?

- [ ] Yes
- [ ] No
- [ ] Not sure

Family History

If YES, who?

- [ ] Biological Grandparent
- [ ] Biological Sibling
- [ ] Biological Father
- [ ] Biological Mother
- [ ] Other

*** Details about the person born with OA/TOF (EA/TEF)**

Was the baby born..?

- [ ] Too early (premature)
- [ ] Too late
- [ ] On time
- [ ] Not sure

*** Does the baby/person born with OA/TOF (EA/TEF) have any of the following associated abnormalities?**

If YES, please check the appropriate box.

If NO, please check NONE

You can choose more than one.

- ☐ Vertebral/Spine abnormalities
- ☐ Anorectal abnormalities (born without normal anus)
- ☐ Heart Problems
- ☐ Kidney or bladder problems
- ☐ Limb problems (arms or legs)
- ☐ Tracheomalacia (floppy windpipe)
- ☐ Chromosomal abnormalities
- ☐ None
- ☐ I am not sure

Questions 6.1 to 7.18 are only for parents/caregivers of children born with OA/EA and/or TOF/TEF completing the survey.

How did you first feel when your child had his/her/their first operation?

You can choose more than one answer.

- ☐ Shocked
- ☐ Emotional
- ☐ Guilty
- ☐ Worried
- ☐ Did not understand the diagnosis
- ☐ None of these

Did they need to transfer the baby to a different hospital for the surgery?

- ☐ Yes
- ☐ No

If YES, was the mother able to go with the baby?

- ☐ Yes
- ☐ No
- ☐ None Of The Above

Was there a place for you to sleep next to your baby?

- [ ] Yes
- [ ] No
- [ ] Not applicable

Could siblings visit the baby?

- [ ] Yes
- [ ] No
- [ ] Not applicable

Could other family visit the baby?

- [ ] Yes
- [ ] No

During pregnancy (for parents of children born with OA/TOF (EA/TEF)):

If the diagnosis was suspected antenatally (while you were pregnant), did anyone offer counselling about the the diagnosis and what to expect after the baby was born?

- [ ] Yes
- [ ] No
- [ ] I can't remember

If YES, do you think this helped you to be better prepared for the baby after the birth?

- [ ] Yes
- [ ] No
- [ ] None Of The Above

Do you think you had the opportunity bonded with your baby during the pregnancy?

- [ ] Yes
- [ ] No
- [ ] None Of The Above

If you feel like you DID NOT bond with the baby during the pregnancy, what do you think was the reason for this?

- [ ] Complicated pregnancy
- [ ] Previously complicated pregnancy
- [ ] First pregnancy
- [ ] Possibility of the diagnosis led to stress
- [ ] Not sure

After the birth (for parents of children with OA/TOF (EA/TEF)):

Did you feel like you missed a chance to bond with your baby?

- [ ] Yes
- [ ] No
- [ ] N/A not the biological parent

If YES, was this because you were unable to pick the baby up in the first few hours/days after birth?

- [ ] Yes
- [ ] No

After the birth, did the doctor/surgeon explain the baby's condition and the road ahead with you?

- [ ] Yes
- [ ] No

Did you find this time was emotional or traumatic?

- [ ] Yes
- [ ] No

Did you have any support from family or friends during this time?

- [ ] Yes
- [ ] No

Did anyone from the hospital offer you any emotional support?

- [ ] Yes
- [ ] No

If yes, who?

You can choose more than one

- [ ] Doctors
- [ ] Nursing staff
- [ ] Councillors
- [ ] Social workers
- [ ] Other parents
- [ ] Other
- [ ] No one

If NO, do you wish that anyone had offered you support?

- [ ] Yes
- [ ] No

Were you in contact with other families with the same condition?

- ☐ Yes
- ☐ No
- ☐ N/A

If NO, do you think this would have helped you during this time?

- ☐ Yes
- ☐ No

Did you have any other children at home during this time?

- ☐ Yes
- ☐ No
- ☐ N/A

If YES, did you feel guilty for neglecting the other children whilst in hospital with your baby?

- ☐ Yes
- ☐ No

Did you ever feel guilty for going home when your baby stayed in hospital/NICU?

- ☐ Yes
- ☐ No
- ☐ Not applicable

If you were in a partnership during this time: How would you describe the impact having a baby with OA/TOF had on your partnership/relationship?

(You may choose more than one)

- ☐ Our partnership was not really affected
- ☐ Our partnership was affected
- ☐ Our partnership was severely affected
- ☐ Our partnership ended
- ☐ We are continuously working on our relationship
- ☐ Our partnership is stronger than ever
- ☐ I was not in a partnership/relationship during this time

*** Emotional trauma and support**

Have you (person completing the form) after your experience caring for a baby with OA/TOF (EA/TEF) ever been diagnosed with any of the following conditions by a healthcare professional (GP, Psychiatrist, Psychologist)?

- [ ] Post-traumatic Stress Disorder (PTSD)
- [ ] Anxiety
- [ ] Depression
- [ ] NO
- [ ] I would rather not say

*** Emotional trauma and support**

Has anyone else in your family been diagnosed with any of the following conditions by a healthcare professional (GP, Psychiatrist, Psychologist) after your experience of caring for a baby with OA/TOF (EA/TEF)?

- [ ] Post-traumatic Stress Disorder
- [ ] Anxiety
- [ ] Depression
- [ ] NO
- [ ] I would rather not say

*** Emotional trauma and support**

Has the person who was diagnosed with OA/TOF (EA/TEF) ever been diagnosed with any of the following conditions by a healthcare professional (GP, Psychiatrist, Psychologist)?

- [ ] Post-traumatic Stress Disorder
- [ ] Anxiety
- [ ] Depression
- [ ] NO
- [ ] I would rather not say

*** Emotional trauma and support**

Do you think sharing your story with others will help YOU?

- [ ] Yes
- [ ] No

*** Emotional trauma and support**

Do you think sharing your story with others will help **THEM?**

- [ ] Yes
- [ ] No

*** Emotional trauma and support**

Do you think an disease specific (OA/TOF, EA/TEF, VACTERL) support group is a good idea?

- [ ] Yes
- [ ] No

Early school development and history

What was the first type of school/care facility you/your child with OA/TOF (EA/TEF) attended?

- [ ] Day mother/father
- [ ] Creche/Nursery school
- [ ] Primary school
- [ ] Home schooled
- [ ] None Of The Above

Early school development/history

If you/your child with OA/TOF (EA/TEF) only attended school at a later stage, what was the reason for this?

- [ ] Fear of illness
- [ ] Fear of lack of understanding the disease
- [ ] Other problems (Neurodevelopmental, feeding tubes, tracheostomy tubes, other)
- [ ] None Of The Above

Early school development/history

If you/your child with OA/TOF (EA/TEF) DID attend school PRIOR to primary school, were there any problems with the following at school?

You can choose more than one.

- [ ] Developmental delays
- [ ] Regular illness
- [ ] Emotional problems
- [ ] Problems with eating
- [ ] None Of The Above

Early school development/history

Did you/your child with OA/TOF (EA/TEF) have any of the following problems in **PRIMARY/MIDDLE** school?

You can choose more than one.

- [ ] Learning difficulties
- [ ] Regular illness
- [ ] Emotional problems (bullying, self-awareness, other)
- [ ] Problems with eating
- [ ] None Of The Above

EAT
Esophageal ATresia
Global Support Groups

Thank you for taking the time to complete this survey.

We hope it would highlight the importance of emotional support for patients and families born with OA/TOF (EA/TEF).
